# Trikresol in Paraurethral Abscess

**Published:** 1903-08

**Authors:** 


### Trikresol in Paraurethral Abscess.

M. L. Heidingsfeld, professor of dermatology, etc., Cincinnati, in a paper read before the North Kentucky Medical Society, November 13, 1902, *(Boston Medical and Surgical Journal,* April 16, 1903), says that many cases of chronic gonorrhea owe their obstinate character and protracted course to the presence of prostatitis, vesiculitis, or cowperitis. But a much larger class owe it to a different and frequently overlooked condition,—an extension of the gonorrheal infection into the numerous small glands, which extensively line the anterior urethra and glans penis and the external and internal surfaces of the prepuce. Their inflammatory and infectious contents are exuded in the form of a molded filament or shred, and often persist long after a urethritis has subsided and the urine has become clear, producing urine loaded with infectious shreds. Careful palpation will reveal their presence as minute indurations. These so-called paraurethral abscesses often become greatly inflamed and considerably enlarged, varying in size from a pea to a pigeon's egg.

The promptness with which these abscesses refilled when freely incised and drained, and their obstinacy, reminded Dr. Heidingsfeld of the large indurated obstinate abscesses which often complicate severe cases of pustular acne, and incited him to try the method which he is successfully using in the latter affection,—namely, hypodermic injections of minute quantities of trikresol into the heart of the abscess. He has used the preparation in all forms of furuncules and localised abscesses, a single injection often completely aborting them in 24 to 48 hours. Its use was then extended to large abscesses and carbuncles with equally gratifying results, and in one instance he believes he aborted an incipient facial erysipelas by injecting it into its emanating point, a small abscess at the outer angle of the eye.

An ordinary 15-minim glass syringe, which is provided with a retaining screw, is filled with trikresol and armed with a 26-guage needle; the air is expressed, and the retaining screw run down until the fraction of a minim can be carefully measured by adjusting the retaining screw. The proper dose, a drop the size of a bead sufficient for the smallest abscess, and a minim or more for the larger ones, is then injected into the center of the abscess.

Prof. Heidingsfeld recounts four cases, of which the following history is typical:

H. S., age 27, had several gonorrheal infections during preceding five years. In June, 1901, several abscesses formed along the anterior border of urethra immediately behind the sulcus coronarius; extirpation was performed, but a mild urethritis persisted and became quite active after several months and was again complicated by abscess formation. October 28, 1901, patient had very active urethritis and two circumscribed abscesses, each about the size of a marble, directly under the line of operative incision. Abscesses were incised and freely drained; but gonorrheal urethritis failed to disappear completely. Abscesses again formed, and were excised and drained on December 22. Improvement was only temporary, and when abscesses appeared a third time, February 17, 1902, they were injected with trikresol. Since then abscesses and urethritis have not reappeared.

In all of the four cases trikresol not only effected a complete cure of the abscesses, but materially cut short what were exceedingly obstinate, refractory and unduly prolonged urethrites.

though four cases scarcely afford sufficient experience to determine the full efficiency of the preparation, yet in connection with the marked success which attends its use in analogous conditions, pustular acne and furunculosis, its ease of administration, the absence of any marked distress or serious complication, thoroughly suffices to warrant its use in every accessible case.

Heidingsfeld also used trikresol in an anomalous and obstinate vulvovaginal abscess, with equally gratifying results:

November 8, Miss I. S., presented herself with a vulvovaginal abscess of the left labium, of the size of a pigeon's egg, tender and sensitive to pressure, accompanied with considerable redness and swelling of the adjacent area. The abscess was incised and a considerable quantity of pus escaped, which on microscopic examination revealed ill-defined, poorly-staining, degenerated pus cells, with an abundance of deeply stained characteristic looking intra- and extracellular gonoccoci. Trikresol injected into center of abscess and an ice-bag applied for twenty-four hours. November 11, patient reported she had experienced scarcely any pain or discomfort from the operation, and that swelling and distress had entirely disappeared. On November 15 the labium had assumed a perfectly normal appearance, and the patient was discharged as cured.

